# Coexistence of Low Coral Cover and High Fish Biomass at Farquhar Atoll, Seychelles

**DOI:** 10.1371/journal.pone.0087359

**Published:** 2014-01-29

**Authors:** Alan M. Friedlander, David Obura, Riaz Aumeeruddy, Enric Ballesteros, Julie Church, Emma Cebrian, Enric Sala

**Affiliations:** 1 Fisheries Ecology Research Lab, Department of Biology, University of Hawaii, Honolulu, Hawaii, United States of America; 2 Coastal Research and Development in the Indian Ocean (CORDIO), Mombasa, Kenya; 3 Island Conservation Society, Mahé, Republic of Seychelles; 4 Centre d'Estudis Avançats de Blanes, Blanes, Spain; 5 UniquEco Designs Ltd, Nairobi, Kenya; 6 National Geographic Society, Washington, DC, United States of America; New England Aquarium, United States of America

## Abstract

We report a reef ecosystem where corals may have lost their role as major reef engineering species but fish biomass and assemblage structure is comparable to unfished reefs elsewhere around the world. This scenario is based on an extensive assessment of the coral reefs of Farquhar Atoll, the most southern of the Seychelles Islands. Coral cover and overall benthic community condition at Farquhar was poor, likely due to a combination of limited habitat, localized upwelling, past coral bleaching, and cyclones. Farquhar Atoll harbors a relatively intact reef fish assemblage with very large biomass (3.2 t ha^−1^) reflecting natural ecological processes that are not influenced by fishing or other local anthropogenic factors. The most striking feature of the reef fish assemblage is the dominance by large groupers, snappers, and jacks with large (>1 m) potato cod (*Epinephelus tukula*) and marbled grouper (*E. polyphekadion*), commonly observed at many locations. Napoleon wrasse (*Cheilinus undulatus*) and bumphead parrotfish (*Bolbometopon muricatum*) are listed as endangered and vulnerable, respectively, but were frequently encountered at Farquhar. The high abundance and large sizes of parrotfishes at Farquhar also appears to regulate macroalgal abundance and enhance the dominance of crustose corallines, which are a necessary condition for maintenance of healthy reef communities. Overall fish biomass and biomass of large predators at Farquhar are substantially higher than other areas within the Seychelles, and are some of the highest recorded in the Indian Ocean. Remote islands like Farquhar Atoll with low human populations and limited fishing pressure offer ideal opportunities for understanding whether reefs can be resilient from global threats if local threats are minimized.

## Introduction

Preserving coral reefs under the threat of climate change presents the difficult challenge of determining how effective conservation efforts will be, when the prognosis for reefs seems so bleak [1]–[3]. Given the facts that nearly all CO_2_ and temperature scenarios exceed current limits of survival of reef corals and many other reef and calcifying species, and adaptation options are essentially unknown and not yet predictable, reefs may disappear on a 50–100 year time scale [1], [4]. This therefore brings into question the utility of conserving reefs at the local scale.

In this paper, we present a scenario for a reef ecosystem where corals may have lost their role as major reef engineers but where fish assemblages are relatively intact. From an ecosystem perspective, the key question is whether the general functions and processes currently attributed to reefs dominated by corals (e.g., high biodiversity, high productivity, and complete food webs with full ecological interactions) can persist with corals playing a lesser role. These functions and processes have value in and of themselves from a perspective of maintaining and conserving mature ecosystems under current circumstances, as well as from the provisioning of some of the ecosystem goods and services to society.

We conducted an extensive assessment of the marine ecosystem of Farquhar Atoll in the Republic of Seychelles in February 2009, in order to provide information for the future protection of this unique and biologically rich atoll. Farquhar Atoll (10°10’S; 51°08’E) lies 770 km south of Mahe, the capital, and is the most southern of the Seychelles islands. Owing to its distance from the capital, no comprehensive marine resource assessment had been conducted prior to this work. While the northern, more populated islands have suffered from local threats (e.g., overfishing, runoff, coastal development) and global threats (climate change) [5]–[7], the more remote areas are still relatively unimpacted by local threats, and offer an important conservation hotspot that requires immediate protection due to the decline of coral reef ecosystems around the world [8]. Furthermore, Farquhar Atoll has been run under a management regime that limits reef fishing to a small local work force (<10 people) and a single tourist operation (primarily catch-and-release), creating a nearly unfished coral reef ecosystem. The purpose of this expedition was to characterize the coral reefs at Farquhar and determine their ecological significance and resilience relative to other reefs in the region and globally, and to make recommendations for protection.

## Methods

The Island Development Corporation of the Republic of Seychelles granted all necessary permission to conduct this research. No vertebrate sampling was conducted and therefore no approval was required by the Institutional Animal Care and Use Committee.

### Site Description

Set in the western Indian Ocean, 1600 km from East Africa, The Republic of Seychelles is situated between 4° and 10° south of the equator and consists of more than 150 islands scattered across the 1,400,000 km^2^ of its exclusive economic zone [5]. The northern, more populated islands are continental and granitic in origin and are a fragment of the continental masses of India and Madagascar, but were isolated from the beginning of the Tertiary (67 million years ago) as India moved northwards opening up the Indian Ocean [9]. The outer islands are comprised of geologically much younger low sand cays on sea-level platform reefs and atolls [10].

**Figure 1:**
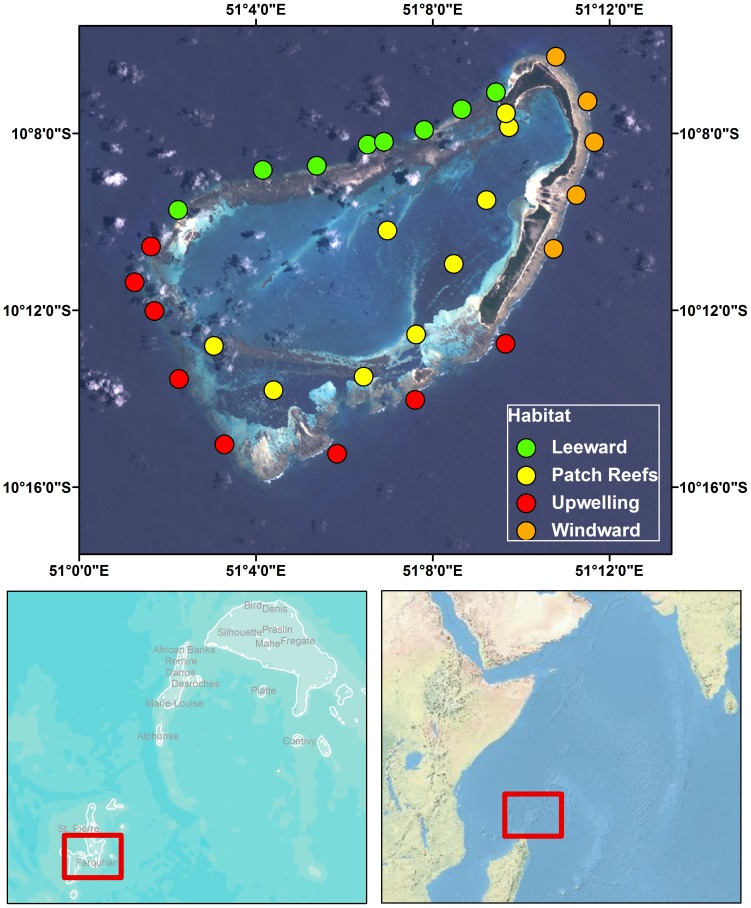
Sampling location at Farquhar Atoll, Republic of Seychelles.

Farquhar Atoll is roughly triangular in shape with a topographically complex lagoon system [11] (Fig. 1). Apart from small sand cays on the northern rim, dry land is confined to the eastern or windward side of the atoll. The lagoon has three main divisions: (1) the main lagoon basin, 17 km long, with the greatest width at 7.5 km; (2) a triangular area on the south side, extending 4.5 km southwards from the rim of the main lagoon basin; and (3) a submerged spur at the northwest corner, extending for 7 km northwestwards with depths of 11–30 m. There is only one entrance to the lagoon, a narrow channel 6–10 m deep near the north point, though water also enters the lagoon over the windward reef flat south of the emergent land and leaves over the leeward reefs [11].

**Integrated ecosystem assessment.** An integrated assessment of fishes, corals, algae, and macro-invertebrates was conducted at Farquhar Atoll in February 2009. Sampling locations were stratified by habitat (fore reef, patch reefs) and wave exposure (windward, leeward, upwelling) and located haphazard within each stratum. The upwelling habitat type was inferred from past studies and datasets that show upwelling on the southern boundary of the atoll and surrounding bank [12] that match local knowledge (R. Aumeeruddy personal observations), and is differentiated from both windward and leeward reef exposures. A total of 33 sites were visited with comprehensive benthic and fish surveys conducted at 30 of these sites between 15 and 27 February 2009.

**Quantitative Surveys of Benthic Cover.** At each site, five 10 m transect lines were deployed with a 10 m gap between each transect. Along each transect, three random photos of 0.5 m^2^ were taken. Benthic cover was classified into major ecological groupings: live coral, dead coral, soft coral, sponges, turf algae, seagrass, erect macroalgae, bare substrate, rubble, encrusting coralline algae, sand, ascidians, and non-coral macro-invertebrates. Coverage was calculated by means of a random point count method with 50 points per photo [13]. Cover for each group at a site was calculated from the grand mean of all transects (N = 750).

**Coral Community Structure.** Coral species were identified by site to provide a comprehensive checklist. Size class distributions for 21 selected common coral genera (*Acanthastrea, Acropora, Astreopora, Coscinaraea, Echinopora, Favia, Favites, Fungia, Galaxea, Goniastrea, Hydnophora, Lobophyllia, Montastrea, Montipora, Pavona, Platygyra, Pocillopora, Porites* (separated into branching and massive morphologies), and *Seriatopora*) were measured [14]. For coral colonies > 10 cm in diameter, size structure of target genera was quantified along 25×1 m belt transects. Sampling of colonies < 10 cm was conducted using six 1 m^2^ quadrats located along the belts at fixed intervals (0, 5, 10, 15, 20, and 25 m). Only colonies whose center was within the transect were counted [14].

**Algae.** Benthic algal cover was quantified *in situ* using 0.5 m^2^ quadrats divided in 25 subquadrats. Fifteen quadrats were positioned haphazardly along the same 10 m transects used to conduct benthic surveys. Algae and other benthic cover within each quadrat were identified to the lowest possible taxonomic level. The coverage of each taxon within each quadrat was estimated by visually assessing coverage in each subquadrat (in 5% cover classes for coverage between 5% and 100% and in 1% cover classes for coverage between 1% and 4%; 0.1% cover was assigned for species with cover < 1%). The sum coverage at some quadrats exceeded 100% due to overgrowth of some taxa on others.

**Fish assemblage surveys.** Fish surveys were conducted by a team of paired divers who enumerated all fishes encountered within fixed-length (25 m) strip transects whose widths differed depending on fish body size (8 m wide for fishes ≥20 cm, 200 m^−2^, and 4 m for fishes <20 cm, 100 m^−2^). While laying the transect line during the ‘swim-out’, divers surveyed adjacent and non-overlapping 4 m wide lanes for larger fishes, focusing observations ahead in a 5 m moving window. The swim-out tally was completed within 3 to 5 min. During the ‘swim-back’, divers quantified the smaller-bodied fishes in adjacent 2 m wide lanes. These transect dimensions were selected to optimize data precision and accuracy, while maximizing cost efficiency of field effort [15]. Constraints on the focal window size and survey duration for the swim-out limited problems of over-counting large-bodied, vagile species. As with any survey method, there are limitations and biases that need to be acknowledged when considering the data. For example, fishes in remote locations are known to be attracted to divers [16] and our sampling method may therefore result in inflated biomass estimates. In addition, visual surveys do not sample cryptic and nocturnal species that make up much of the biodiversity of the reef [17].

The species identity and visually estimated length (in 5 cm increments of total length - TL) were recorded for each individual fish. Cryptic species and individuals <3 cm TL were not tallied. Two to three transects, separated by ca. 5 m, were completed by each diver pair at each station covering a linear reef extent of between 55 and 90 m.

Length estimates of fishes from censuses were converted to mass (M) using the following length–mass relationship: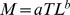, where the parameters a and b are constants for the allometric growth equation and TL is total length in cm [18]. All biomass estimates were converted to metric tonnes per hectare (t ha^–1^) and numerical abundance estimates were converted to number of individuals m^−2^. Fish species diversity was calculated from the Shannon- Weaver diversity index [19]: 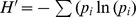), where *p_i_* is the proportion of all individuals counted that were of species *i*. Fishes were categorized into four trophic groups (piscivores, herbivores, invertivores, and planktivores) after [20]-[21].

**Surveys of large resource fishes.** Large resource fishes were surveyed at each site by one diver swimming across the survey site. During a period of 45 minutes all large fishes (> 30 cm) were counted in a 10 m wide belt (5 m on each side of the diver). Transect length varied depending on current and reef topography.

**Statistical methods.** Nonmetric multidimensional scaling (nMDS) analysis using PRIMER v5 [22] was conducted to examine benthic community structure among habitats. The data matrix consisted of percent cover for each major cover type (live coral, soft coral, turf algae, macroalgae, crustose coralline algae - CCA, sand, seagrass). A Bray-Curtis similarity matrix was created from the arcsine square root transformed cover matrix prior to conducting the nMDS. A permutation-based hypothesis testing analysis of similarities (ANOSIM in PRIMER 5.0, Primer-E Ltd., Plymouth, UK) was used for the comparison of hard-bottom benthic assemblages among habitat types [22]–[23]. This procedure generates an R statistic on a scale from 0 or negative value (identical assemblages) to 1 (completely dissimilar assemblages). The resulting P value indicates the probability that the two assemblages come from a similar distribution [23]. The R statistic represents pairs that are well separated (R>0.75), overlapping but clearly different (R>0.5), or barely separable at all (R<0.25).

Benthic cover among major functional groups (e.g., live coral, turf algae, macroalgae, CCA) within each major habitat exposures (e.g., leeward, windward, patch reefs, upwelling) was compared using one-way analysis of variance (ANOVA). Unplanned comparisons between pairs were examined using the Tukey-Kramer HSD (honestly significant difference) test for ANOVAs (α = 0.05). The number of coral recruits (≤ 5 cm colony size) among habitats was compared in a similar manner. Fish assemblage characteristics (e.g., species richness, number of individuals, biomass, and diversity) were also compared among habitat exposures using one-way ANOVA and Tukey-Kramer HSD. Number of individuals (no. m^−2^) and biomass (t ha^−1^) were ln(x+1)-transformed prior to statistical analysis to conform to the assumptions of parametric statistics [24]. Normality was tested using a Shapiro-Wilk W test (P = 0.05) while a Bartlett’s test (P = 0.05) was used to examine homogeneity of variance. Fish trophic biomass data did not conform to parametric statistical assumptions despite various transformations, therefore biomass comparisons within and among habitat exposures were conducted using a Kruskal-Wallis rank-sum test (H) with Dunn’s test for unplanned multiple comparisons. The Island Development Corporation of the Republic of Seychelles granted all necessary permission to conduct this research. No vertebrate sampling was conducted and therefore no approval was required by the Institutional Animal Care and Use Committee.

## Results

### Benthic Community Structure

**Figure 2:**
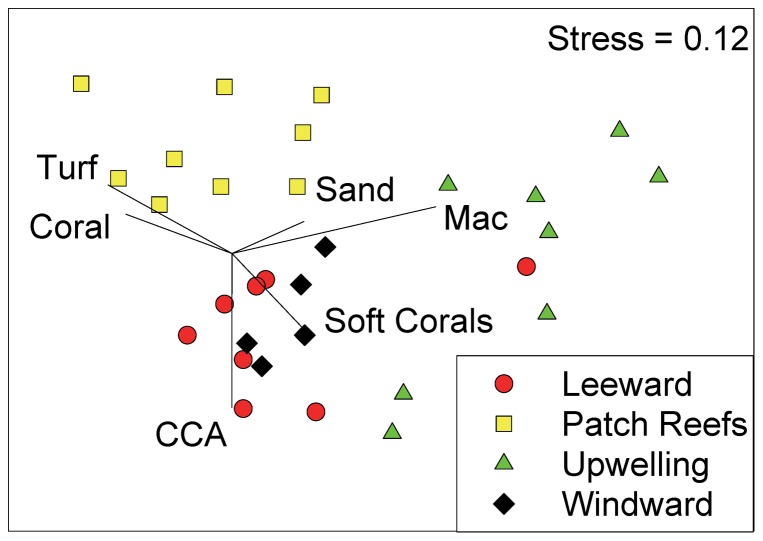
Non-metric multidimensional scaling plot of benthic cover type by location. Values were arcsine squareroot transformed prior to analysis. Vectors are the relative contribution and direction of influence of benthic components to the observed variation among sites. Only benthic components with a correlation > 0.3 with either axis MDS1 or MDS2 were selected for representation in the vector plot.

Viewed in ordination space, stations separated well into the *a priori* habitat types based on benthic community components (Stress  =  0.12, Fig. 2). There was high concordance within patch reef sites, while the other habitats showed somewhat greater variability. There were significant differences in benthic community structure among all habitat types (ANOSIM Global R = 0.56, p<0.001) and pair-wise comparisons among habitat types found benthic communities in the patch reef and upwelling habitats to be most dissimilar (R = 0.83). All other pair-wise comparisons showed clear differences between habitats (all R > 0.5) except for the leeward and windward habitats, which were indistinguishable in benthic community structure (R = 0.11). Turf algal and live coral were most closely correlated with the patch reef habitats, while erect macroalgae were strongly correlated with upwelling areas. Crustose coralline algae (CCA) were highly correlated with leeward habitats with soft coral most closely correlated with windward locations.

**Figure 3:**
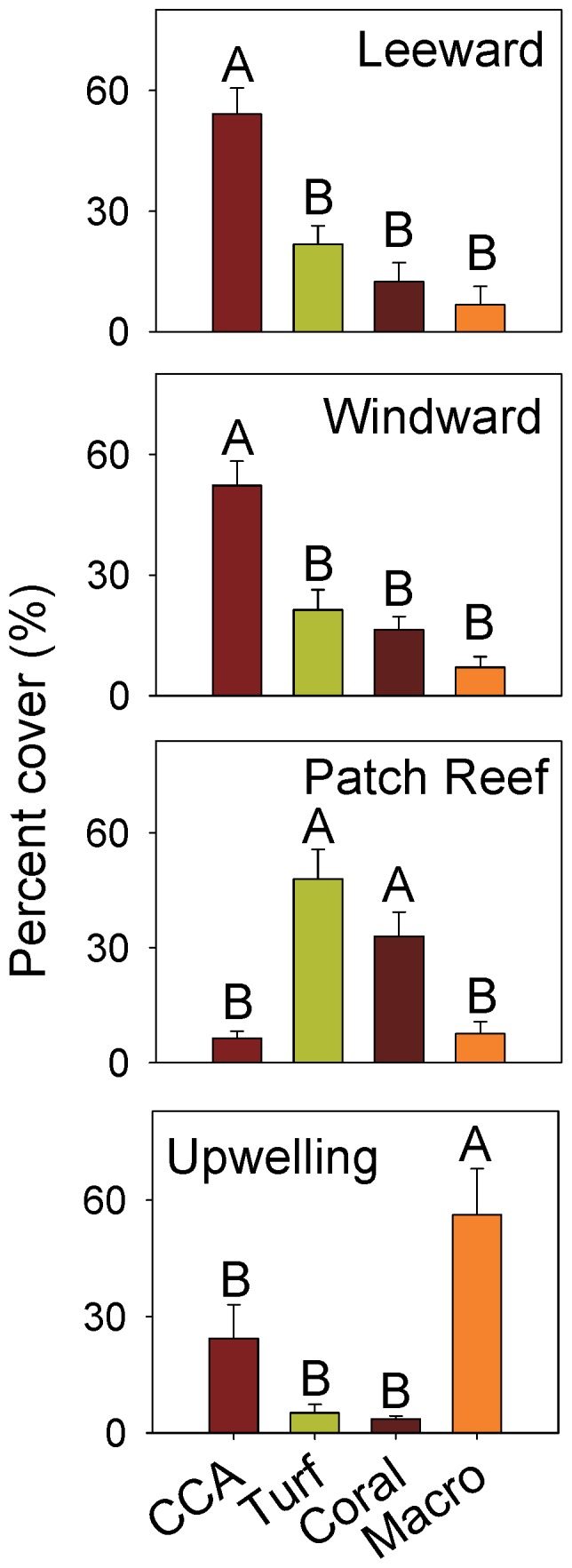
Comparisons of benthic cover among habitat types. CCA, encrusting coralline algae; Turf, turf algae; Coral, live coral; Macro, erect macroalgae. ANOVA results in text. Habitats with the same letter are not significantly different based on Bonferroni-corrected multiple comparisons tests. Error bars area standard error of the mean.

CCA was the dominant benthic cover overall (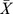 = 31.6%±26.2 sd), followed by turf algae (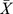 =  25.3%±22.1 sd), erect macroalgae (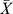 = 20.2%±28.8 sd), and live coral (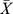 =  16.9%±16.9% sd). CCA was the dominant benthic cover type in both the leeward and windward habitat types and was significantly higher than the other major benthic cover types on both habitats (F_3,31_ = 17.0, p<0.001, F_3,19_ = 19.5, p<0.001, respectively Fig. 3). Patch reef habitats were dominated by turf and live coral, both of which had significantly larger cover than CCA and erect macroalgae (F_3,35_ = 14.6, p<0.001, Fig. 3). Erect macroalgae dominated at the upwelling sites and had significantly higher cover than the other major benthic groups (F_3,51_ = 10.6, p<0.001, Fig. 3).

### Coral Diversity

A total of 140 species and 44 genera of scleractinian corals were identified at Farquhar during the survey with the highest number of species per genera recorded for *Montipora* (11), *Favites* (10) and *Acropora* (10). The genera *Porites* and *Montipora* were overwhelmingly dominant on the atoll, followed by *Favia*, *Favites*, *Acropora* and *Goniastrea*. Area coverage was dominated by *Porites* (23%) and *Montipora* (22%), followed by *Acropora* (13%) by area coverage, while *Porites* (15%), *Pocillopora* (14%), and *Montipora* (13%) were the most common genera by number of colonies present.

### Site-level benthic composition

**Figure 4:**
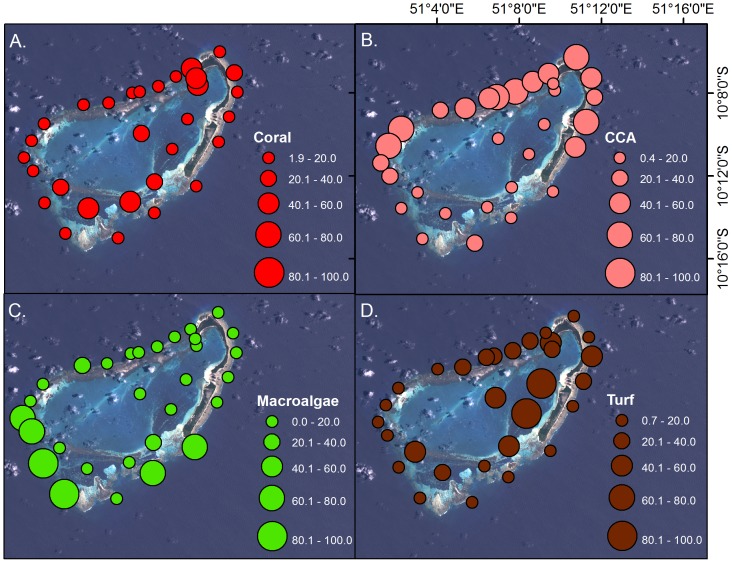
Site-level benthic cover by major group. A. Live coral, B. Crustose coralline algae (CCA), C. Macroalgae, D. Turf algae.

Coral cover was highest near the channel at the northern end of the atoll, as well as the southern portion of the lagoon (Fig. 4). The cover of CCA was high around much of the outer portion of the atoll, with low cover in the lagoon and the southern forereef. Macroalgal cover was high round the southern portion of the forereef but low elsewhere. Turf algae was most abundant on the lagoonal patch reefs.

### Coral Colony Size Structure

**Figure 5:**
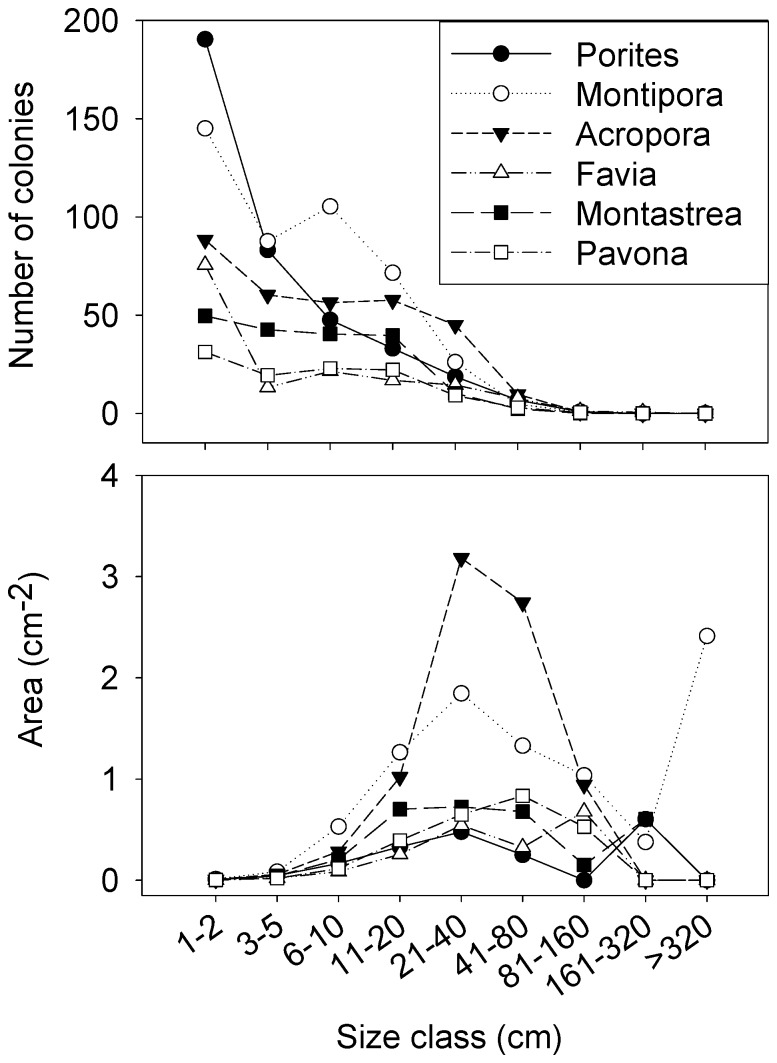
Size class distribution of the most abundant coral genera. A. Size class distributions by total area, B. Size class distributions by number of individuals 100 m^−2^.

More than 76% of the coral colonies sampled were within the three smallest size classes (< 10 cm, Fig. 5a). However, the 20–40 cm size class dominated the coral populations by area (Fig. 5b), in contrast to many mature reefs where the two largest size classes (1.6–3.2 m and > 3.2 m) typically dominate the area distribution [25]–[26]. The number of coral recruits (≤ 5 cm colony size) averaged 14.1 (± 12.4 sd) individuals m^−2^ and varied significantly among habitat types (F_3,29_ = 4.6, p = 0.01). The highest number of recruits was observed along the leeward slope (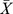 =  25.6±17.1), with the lowest occurring in the upwelling region (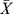 =  7.1±9.1). This was the only habitat comparison where the number of recruits differed significantly (p<0.5) despite two-fold differences in recruit size between the leeward vs. patch reef (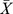
_ = _ 12.4±5.5) and windward (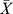
_ = _ 9.8±3.9) habitats.

### Macroalgal diversity

A total of 54 macroalgal taxa were recorded at Farquhar during the survey, which included 29 greens, 18 reds, 7 browns, 2 cyanobacteria, and 1 seagrass (Table S1). Red algae made up 19.6% of the total benthic cover but were highly variable in abundance (± 25.0% sd). Brown macroalgae accounted for an additional 8.8% (± 16.5% sd) of the total benthic cover, followed by green algae (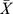 =  6.1%±9.4% sd), and cyanobacteria (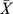 =  0.3%±0.5% sd). Red and brown macroalgae were the most common groups in the leeward and upwelling habitats. Green macroalgae was most abundant in windward habitats, while brown and green macroalgae were most common in the patch reef habitats.

The crustose coralline *Hydrolithon onkodes* was the dominant benthic cover in windward and leeward forereef habitats, but had low cover on the lagoonal patch reefs (Table 1). The brown macroalga *Lobophora variegata* was the next most abundant species overall and was common in all habitat types. The red macroalgae *Peyssonnelia* spp. were abundant in the windward and leeward habitats but were present in low abundance in the other habitat types. In the upwelling habitat, the red alga *Botryocladia skottsbergii* was the second most abundant macroalga following *Lobophora*. The green algae *Boodlea struveoides* and *Microdictyon okamurae* were the most abundant macroalgae within the windward habitat type.

**Table 1:** The top 20 species of macroalgae observed on quantitative transects at Farquhar. Values are mean percentage cover (± sd). Species are listed in descending order by total cover.

Group	Species	Total	Leeward	Windward	Upwelling	Patch reefs
Red	*Hydrolithon onkodes*	23.4 (21.5)	39.2 (20.3)	49.6 (5.1)	11.7 (9.6)	5.3 (6.6)
Brown	*Lobophora variegata*	4.4 (7.9)	3.6 (2.5)	1.5 (2.5)	4.4 (6.2)	6.9 (13.0)
Brown	Unidentified leather brown alga	4.2 (15.2)	6.1 (17.1)	-	9.8 (24.4)	-
Red	*Peyssonnelia* spp.	3.1 (4.8)	6.8 (7.6)	4.3 (1.1)	0.8 (1.1)	1.1 (2.4)
Red	*Botryocladia skottsbergii*	2.4 (9.7)	-	-	8.9 (18)	-
Green	*Halimeda opuntia*	1.6 (3.2)	2.3 (2.9)	0.3 (0.4)	2.3 (5)	1.0 (2.4)
Green	*Boodlea struveoides*	1.1 (3.8)	0.9 (2)	5.0 (8.6)	0.1 (0.2)	<0.1 (0.1)
Green	*Caulerpa racemosa*	0.9 (4.5)	-	-	-	3.1 (8.1)
Green	*Microdictyon okamurae*	0.8 (2.5)	0.2 (0.4)	4.6 (5.0)	-	-
Red	*Sporolithon ptychoides*	0.8 (2.9)	<0.1 (0.1)	0.2 (0.2)	2.9 (5.3)	-
Green	*Caulerpa cupressoides*	0.6 (2.4)		3.6 (5.2)	0.2 (0.5)	-
Red	*Mesophyllum* sp.?	0.6 (1.4)	1.0 (2)	2.1 (1.3)	-	-
Red	*Amphisbetema indica*	0.5 (1.6)	0.4 (1)	-	1.6 (2.7)	-
Green	*Chlorodesmis fastigiata*	0.4 (1.1)	-	-	0.3 (0.7)	1.0 (1.9)
Red	*Galaxaura rugosa*	0.3 (1.2)	0.8 (2.1)	-	0.4 (1.0)	-
Red	*Hydrolithon gardineri*	0.2 (1.0)	-	1.5 (2.3)	-	-
Green	*Valoniopsis pachynema*	0.2 (0.8)	-	0.1 (0.1)	0.8 (1.4)	-
Green	*Caulerpa serrulata*	0.2 (0.3)	0.1 (0.1)	-	0.2 (0.4)	0.3 (0.3)
Red	*Hypnea pannosa*	0.1 (0.4)	-	-	0.4 (0.7)	-
Green	*Neomeris van bosseae*	0.1 (0.7)	<0.1 (0.1)	<0.1 (0.1)	0.3 (0.7)	<0.1 (0.1)

### Fishes

A total of 372 fish taxa from 56 families were encountered on quantitative surveys around Farquhar Atoll during the expedition. An average of 32.9 (± 7.1) species were observed per transect with no significant difference in species richness among the four habitat types (F_3,29_ = 0.7, p = 0.6). The number of individuals was similar among habitat exposures (F_3,29_ = 1.3, p = 0.3), as was diversity (F_3,29_ = 0.21, p = 0.9). Biomass averaged 3.2 t ha^−1^ (±2.8 sd) overall and was highest in the upwelling habitat (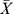
_ = _ 3.7±4.5) and lowest in the patch reef habitat (2.5±1.6), although these differences were not significant (F_3, 29_ = 0.4, p = 0.7).

The red snapper, *Lutjanus bohar*, was the most abundant fish species by mass, accounting for 8.0% of the total biomass and occurring at 73% of all stations, and was most abundant in the leeward habitat (Table 2). The second most abundant species by weight was the economically important, and IUCN Red listed (vulnerable) bumphead parrotfish (*Bolbometopon muricatum*), which accounted for 6.8% of the total biomass and was present at 13% of the stations. This species was most abundant in the upwelling areas but virtually absent in the patch reef and windward habitats. Bluefin trevally (*Caranx melampygus*) ranked third overall in weight, accounted for 6.2% of the total assemblage biomass, and occurred at 70% of all stations. This was followed by another IUCN Red listed (vulnerable) species, the Napoleon wrasse (*Cheilinus undulatus*), which contributed 6.1% of the total biomass and was present at 53% of the stations. It was common in all habitats except for the patch reefs, where it was found in low abundance. The Dark-banded fusilier (*Pterocaesio tile*), ranked fifth overall, accounted for 5.8% of the total biomass and was present at 60% of the stations. The top fifteen species in Table 2 accounted for 62% of the total assemblage biomass.

**Table 2:** Top fish species observed on transects by average (± sd) mass (t ha^−1^) and listed in descending order by average biomass overall. N = 30, n = 88.

Common name	Species	Total	Freq.	Leeward	Patch Reef	Upwelling	Windward
Red Snapper	*Lutjanus bohar*	0.26 (0.48)	73.3	0.36 (0.88)	0.20 (0.36)	0.24 (0.69)	0.25 (0.42)
Bumphead parrotfish	*Bolbometopon muricatum*	0.22 (0.84)	13.3	-	0.05 (0.26)	0.71 (2.83)	0.12 (0.48)
Bluefin trevally	*Caranx melampygus*	0.21 (0.24)	70.0	0.18 (0.34)	0.27 (0.54)	0.10 (0.23)	0.27 (0.44)
Napoleon wrasse	*Cheilinus undulatus*	0.20 (0.27)	53.3	0.20 (0.57)	0.06 (0.17)	0.25 (0.57)	0.38 (0.61)
Dark-banded fusilier	*Pterocaesio tile*	0.19 (0.31)	60.0	0.40 (0.50)	<0.01 (0.03)	0.10 (0.16)	0.35 (0.38)
Paddletail snapper	*Lutjanus gibbus*	0.17 (0.31)	56.7	0.07 (0.28)	0.12 (0.37)	0.16 (0.55)	0.43 (0.52)
Marbled coral grouper	*Plectropomus punctatus*	0.16 (0.22)	66.7	0.18 (0.26)	0.21 (0.53)	0.08 (0.21)	0.17 (0.24)
Potato cod	*Epinephelus tukula*	0.11 (0.28)	23.3	0.07 (0.24)	-	0.29 (0.60)	0.12 (0.35)
Golden trevally	*Gnathanodon speciosus*	0.08 (0.44)	3.3	-	-	0.31 (1.49)	-
Camouflage grouper	*Epinephelus polyphekadion*	0.08 (0.14)	46.7	0.10 (0.22)	0.16 (0.24)	0.01 (0.04)	0.01 (0.03)
Steep-head parrotfish	*Chlorurus strongylocephalus*	0.06 (0.12)	46.7	0.07 (0.13)	01.2 (0.28)	-	0.06 (0.07)
Onespot snapper	*Lutjanus monostigma*	0.06 (0.21)	26.7	0.15 (0.47)	0.01 (0.04)	0.06 (0.29)	0.03 (0.10)
Bigeye emperor	*Monotaxis grandoculis*	0.06 (0.13)	56.7	0.12 (0.45)	0.05 (0.07)	-	0.06 (0.12)
Spangled emperor	*Lethrinus nebulosus*	0.06 (0.19)	26.7	0.01 (0.03)	0.05 (0.11)	0.14 (0.36)	0.03 (0.08)
Oriental sweetlips	*Plectorhinchus vittatus*	0.05 (0.17)	20.0	0.08 (0.20)	-	0.13 (0.55)	0.01 (0.05)

**Figure 6:**
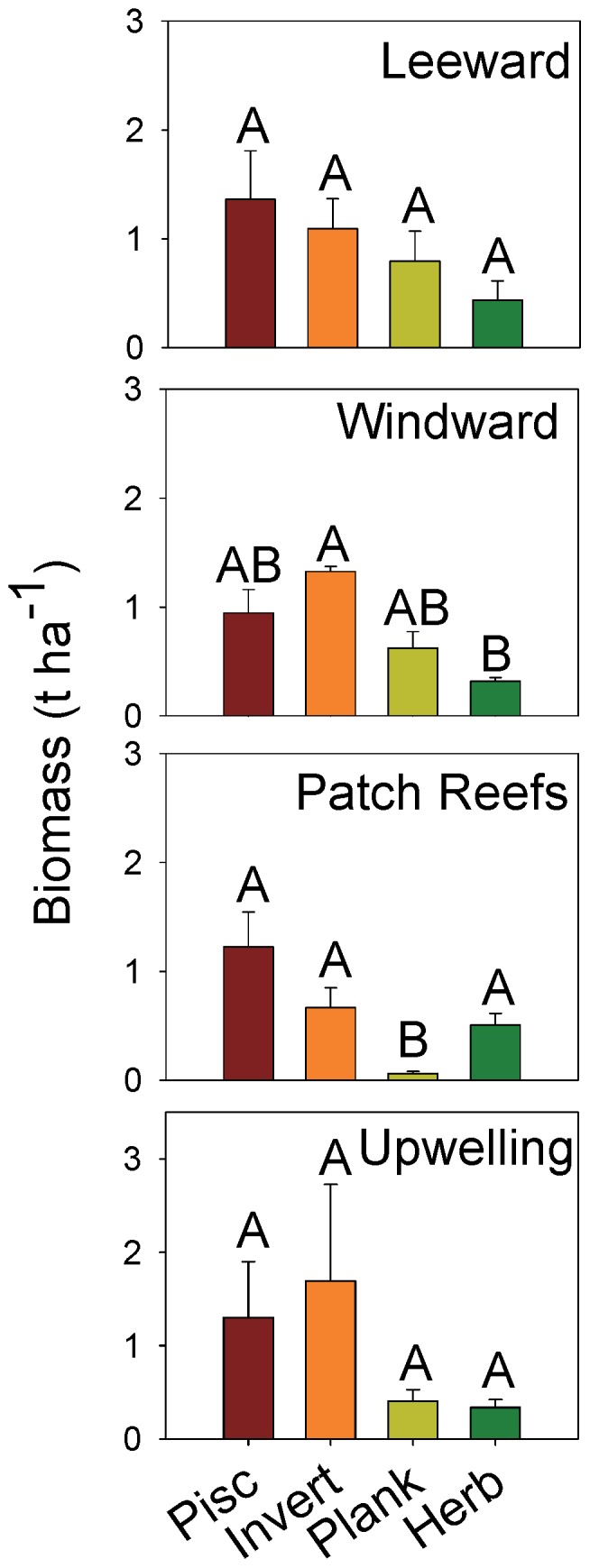
Fish trophic biomass (t ha^−**1**^) by habitat type. Pisc – piscivores, invert – invertivore, plank – planktivore, herb – herbivore. Trophic groups with the same letter are not significantly different based on Tukey-Kramer HSD (honestly significant difference) test for unplanned comparisons. Error bars area standard error of the mean.

Piscivores and invertivores each made up roughly 38% and 36%, respectively of total fish biomass at Farquhar, followed by planktivores (14%) and herbivores (13%) (Fig. 6). Piscivores were most abundant in the leeward and upwelling areas but not significantly different among exposures (H = 0.6, p = 0.9). This trophic group was dominated by red snapper, bluefin trevally, and the marbled coral grouper (*Plectropomus punctatus*). More than 19% of the invertivore biomass was comprised of bumphead parrotfish, followed by Napoleon wrasse (17%) and paddletail snapper (15%). Despite biomass being 2.5 times higher in the upwelling areas compared with the patch reef habitats, the high variability in the schools of bumphead parrotfish resulted in no significant difference among exposures (H = 4.2, p = 0.2). Planktivores were most abundant in the leeward habitats and virtually absent on the lagoonal patch reefs (H = 15.0, p = 0.002). The dark-banded fusilier comprised 43% of the biomass in this trophic group. Herbivores were evenly distributed among habitats (H = 1.3, p = 0.7), with the steephead parrotfish (*Chlorurus strongylocephalus*) and the greenthroat parrotfish (*Scarus prasiognathos*) accounting for 16% and 13% of the biomass in this trophic group, respectively.

Piscivores were the most important trophic group by biomass (37% of total) in the leeward habitats, followed by invertivores (30%), planktivores (21%), and herbivores (12%), although differences were not significant (H = 5.6, p = 0.1). Invertivores were most abundant by biomass (41%) in the windward exposures and were significantly different than herbivores, which made up only 10% of the biomass in this habitat (H = 13.1, p = 0.005). Patch reefs were dominated by piscivores (50% of total) with planktivores virtually absent from this habitat type (3% of total, H = 20.5, p<0.001). Invertivores and piscivores made up the majority of the biomass in the upwelling habitat (41% and 35%, respectively), but the high variance of invertivores, primarily bumphead parrotfish, results in no differences among trophic groups in this habitat (H = 4.2, p = 0.2).

### Site-level fish trophic structure

**Figure 7:**
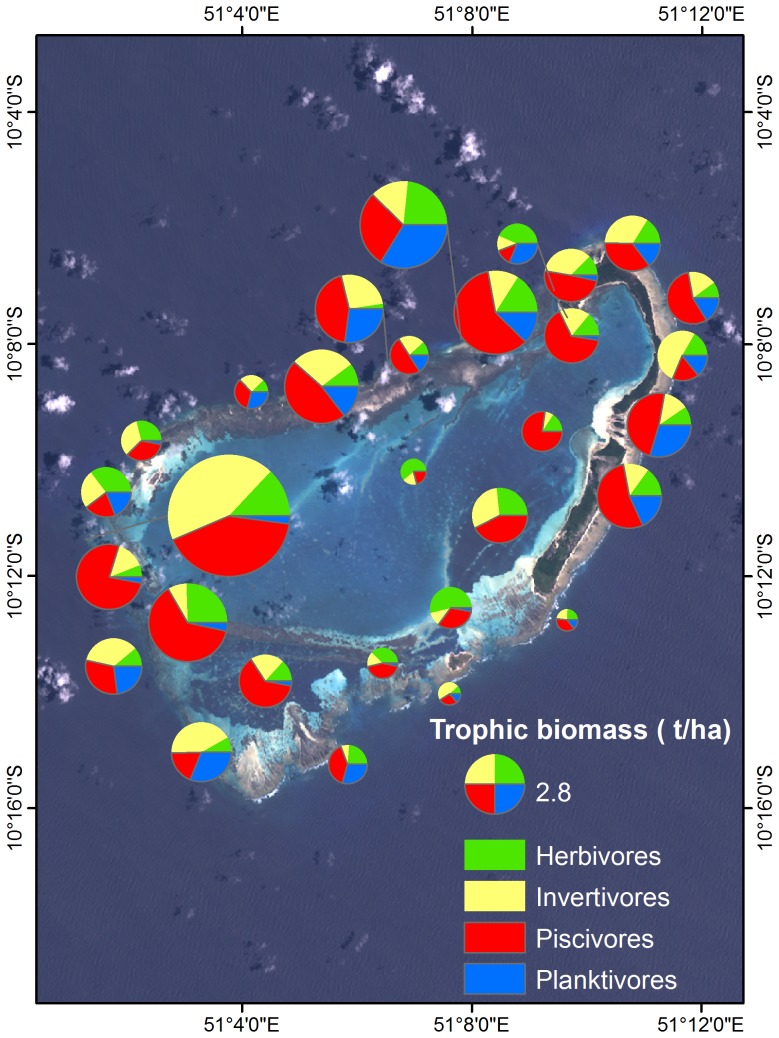
Site-level fish trophic structure by biomass (t/ha). Pie size is proportional to total biomass at each location.

Overall, fish biomass was highest along the leeward (western) forereef, with piscivores also being most abundant in these locations (Fig. 7). Planktivores were common around the outside of the atoll but were virtually absent in the lagoon. Invertivores and herbivores occurred across all locations without pattern.

### Surveys of Large Fishes

A number of large and highly prized resource species were commonly encountered on long swims around the atoll (Table 3). Napoleon wrasses were observed at 86% of the survey sites at Farquhar, with more than five individuals per transect, and ranged in size from 50 cm juveniles to nearly 2 m adult males. Bumphead parrotfish were observed at 36% of the sampling locations at Farquhar with one school in excess of 600 individuals. The marbled coral grouper was the most common grouper species observed, occurring at 76% of the sites, followed by the camouflage grouper, which was observed at 67% of the stations.

**Table 3:** Abundance (mean ± sd per transect), maximum number per transect (N max.) and percent frequency of occurrence (Freq. occur.) of large, resources species from long swim surveys conducted around Farquhar Atoll. N = 33. Taxa are ordered phylo-genetically.

Common name	Creole name	Species	Abundance	N max.	% Freq. occur.
**Groupers (Serranidae)**					
Camouflage grouper	Vyey goni	*Epinephelus polyphykedion*	2.25 (2.13)	8	66.7
Potato cod	Vyey toukoula	*Epinephelus tukula*	0.72 (1.42)	7	36.4
Brown-marbled grouper	Vyey masata	*Epinephelus fuscoguttatus*	0.25 (0.92)	5	12.1
White-blotched grouper	Vyey plat	*Epinephelus multinotatus*	1.31 (1.82)	7	51.5
Blacksaddled coral grouper	Vyey babonn zonn	*Plectropomus laevis*	1.28 (3.32)	18	33.3
Marbled coral grouper	Babonn	*Plectropomus punctatus*	6.13 (8.83)	38	75.8
Yellow-edged lyretail	Krwasan	*Variola louti*	2.63 (4.39)	18	48.5
**Snappers (Lutjanidae)**					
Green jobfish	Zob gri	*Aprion virescens*	0.94 (2.72)	15	27.3
Two-spot snapper	Varavara	*Lutjanus bohar*	14.31 (28.19)	126	72.7
**Grunts (Haemulidae)**					
Oriental sweetlip		*Plectorhinchus vittatus*	1.63 (6.89)	39	21.2
**Emperors (Lethrinidae)**					
Spangled emperor	Kapten rouz	*Lethrinus nebulosus*	6.19 (24.03)	135	33.3
**Jacks (Carangidae)**					
Bluefin trevally	Karang ver	*Caranx melampygus*	13.38 (18.73)	63	72.7
**Wrasses (Labridae)**					
Napoleon wrasse	Aya Gerar	*Cheilinus undulatus*	5.16 (4.46)	15	81.8
**Parrotfishes (Scaridae)**					
Bumphead parrotfish	Filanbaz	*Bolbometopon muricatum*	21.56 (110.94)	629	42.4

### General Oceanography and Geomorphology

**Figure 8:**
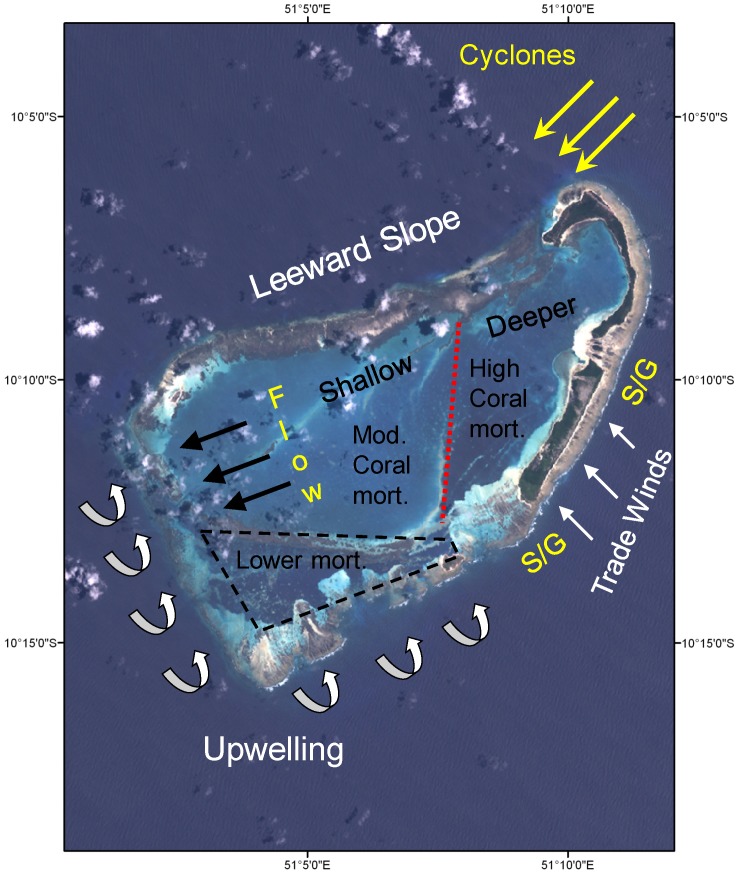
Conceptual model of oceanography and geomorphology of Farquhar Atoll.

Based on survey results and knowledge of the region, we developed a conceptual model of the general oceanography and geomorphology of Farquhar Atoll (Fig. 8). The windward facing, eastern shoreline is typified by low relief spur and groove reef formation with a gradual slope down to approximately 20 m. The leeward slope is steeper with low coral cover but a high cover of crustose coralline algae. The southern portion of the atoll had a lower water temperature than the other portions of the atoll during our surveys and was dominated by a higher cover of macroalgae. Based on these observations and previously published information [12], localized upwelling appears to be a common feature in this region. Previous coral mortality, assumed to be associated with the 1998 El Niño event, was highest in the deeper eastern lagoon, where circulation was limited. The general circulation pattern within the lagoon appears to flow from the east and out the western portion of the atoll. The lowest coral mortality and highest cover was in the southwestern portion of the atoll.

## Discussion

There is a general sequence of coral reef degradation worldwide that involves removal of the large animals (e.g., sharks and large predators), followed by overfishing of animals lower in the food chain (e.g., parrotfishes) and later, usually followed by a time lag, the decline of corals [8], [27]-[28]. Our results show that Farquhar Atoll deviates from this general pattern. While sharks are rare at Farquhar, other large consumers are very abundant, including large groupers, wrasses and parrotfishes. However, coral cover is very low, relative to pristine reefs elsewhere [21], [29], although the three-dimensional structure provided by dead corals is still present. Could Farquhar be a glimpse of future coral reef no-take reserves where fishes are safe from exploitation but corals suffer from global impacts such as global warming?

Like many other areas around the world, the Seychelles has experienced serious declines in coral reef ecosystem health, particularly around the populated islands where local impacts such as overfishing act in synergy with global impacts such as warming events [7], [30]. Remote islands like Farquhar with low human populations and limited fishing pressure offer ideal opportunities for understanding whether reefs can be resilient from global threats if local threats are reduced.

Coral cover and overall benthic community condition at Farquhar was poor, likely due to a combination of factors. The atoll is relatively isolated from the larger areas of the Mascarene plateau, Madagascar, and the East African mainland, and is subject to equatorial upwelling along its southern edge, reducing habitat quality for coral growth. It sits on a broad shallow plateau, and lacks the steep slopes that provide diverse habitat for corals. The flat plateau also results in high sediment movement across reef surfaces, reducing habitat quality for corals. Extensive mortality of corals in the past was evident, particularly in the lagoon, with large dead colonies and clear mortality of small corals. The dead skeletons were highly eroded but in place, suggesting mortality around the time of the 1998 mass mortality of corals in the western Indian Ocean. More recent mortality may be a result of the 2005 bleaching event, which was more severe in the southern Indian Ocean compared with the 1998 event [31]-[32]. At 10°S, Farquhar is at the edge of the cyclone belt, and had a narrow miss of a cyclone in 2007, which hit Providence/Cerf slightly to the north. The northwest slopes of Farquhar show clear cyclone rubble, partially consolidated on the reef slope, but with high levels of disturbance limiting the growth and survival of corals. Overall, this results in a relatively poor coral community, with relatively high coral cover mainly limited to a narrow 5-10 m belt on the W, S, and E slopes of the atoll, and a broader belt down to 25 m on the N-NW slopes. The coral communities in high-flow areas of the lagoon (main channel in the northern, and the SE and SW lagoons) also tended to have relatively higher coral cover.

Coral species richness at Farquhar (n = 140) is among the lowest recorded for the Western Indian Ocean [33], and only comparable to the more remote Mascarene Islands (Reunion and Mauritius), which have a total coral fauna of some 160 species [33]–[34]. The only site with fewer corals is Tromelin Island, between Reunion and Madagascar at about 12°S, which has <40 species as a result of its isolation and a very small size. The dominant size class of corals is the 20–40 cm size class, principally of *Montipora* and *Porites*, indicating past mortality of the larger size classes, and perhaps 5–8 years of growth.

Dominance of *Porites* (massive) and *Montipora* in both small (by numbers) and large (by area) size classes suggest the coral community at Farquhar is somewhat stable, though the lack of large colonies from 40–160 cm suggests significant mortality of these normally abundant corals in the past, very likely due to a large scale event such as the mass bleaching of corals in 1998 and/or 2005. With the exception of the southern upwelling area of the atoll, cover of non-crustose macroalgae was relatively low, likely due to high herbivory and grazing pressure by the high biomass of parrotfishes. Large parrotfishes perform a variety of ecosystem functions in addition to algal grazing, including sediment removal, bioerosion and coral predation that are critical to ecosystem function [35], [36]. This, coupled with the high cover of coralline algae, implies a potentially resilient reef ecosystem, yet one with low coral cover. The coral reef of Farquhar is thus different from the inner Seychelles, which have undergone a widespread phase shift from a coral-dominated state to rubble and algal-dominated states [30]. These inner reefs on the Mahe Plateau were dominated by highly complex branching corals with low herbivore biomass in most places, while the coral assemblage at Farquhar consists primarily of massive and encrusting species that are more resistant to bioerosion. At Farquhar, macroalgae have been kept in check by the extraordinary abundance of herbivorous fishes. The patch reefs of the lagoon are likely to continue to erode and degrade over the coming years given the current levels of recruitment and projections for climate change. However the species composition of the outer reef consists of slower-growing genera with massive or encrusting growth forms that are less susceptible to bleaching, and may therefore be more resilient to climate change compared to more susceptible species such as branching *Acropora* [37]. Rates of reef degradation may show high spatial and temporal variability ranging from complete collapse of coral cover to maintenance of comparable levels of cover to 2100 and beyond [38]. We therefore predict that the topographic complexity in the lagoon at Farquhar may decline over the next several years while the forereef habitat may remain resilient for decades or even longer.

**Figure 9:**
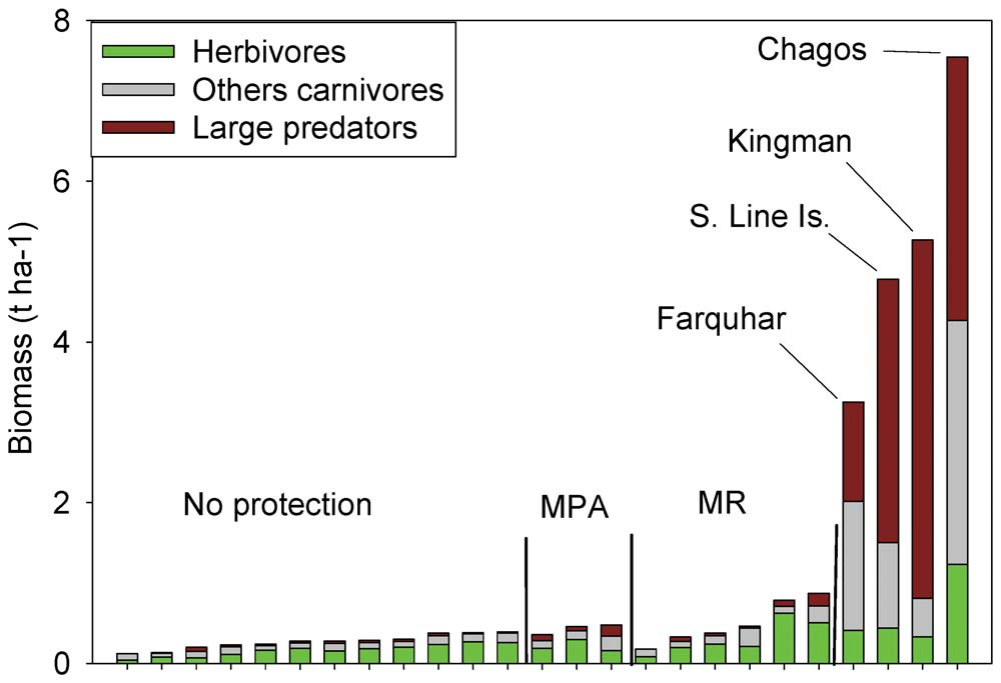
Comparisons of fish biomass by trophic group among various management regimes in the Seychelles and pristine reefs in the Indo-Pacific. MPA  =  marine protected area, MR  =  no-take marine reserves. Data from [7], [21], [30], [39], [40].

Farquhar Atoll harbors a relatively intact reef fish assemblage structure reflecting ‘natural’ ecological processes that are not influenced by fishing or other local anthropogenic factors. The most striking feature of the reef fish assemblage at Farquhar is large biomass, which is nearly an order of magnitude greater than at unprotected locations around the northern Seychelles, and also much higher than even within marine protected areas and no-take reserves in the Seychelles (Fig. 9, [7], [30]). Differences in methodologies may account for some of these observed patterns but the stark contrasts highlight the effectiveness of protecting whole ecosystems with minimal local impacts compared to the relatively small protected areas that currently exist in the more populated northern Seychelles. In fact, this biomass is among the highest recorded in the Indian Ocean although the absence of reef sharks clearly distinguishes Farquhar from more pristine locations in the region such as the Chagos Archipelago [39]–[40]. Comparisons with remote, unfished locations in the Line Islands using the same sampling methodology [20] show Farquhar with comparable biomass but with fewer top predators.

**Figure 10:**
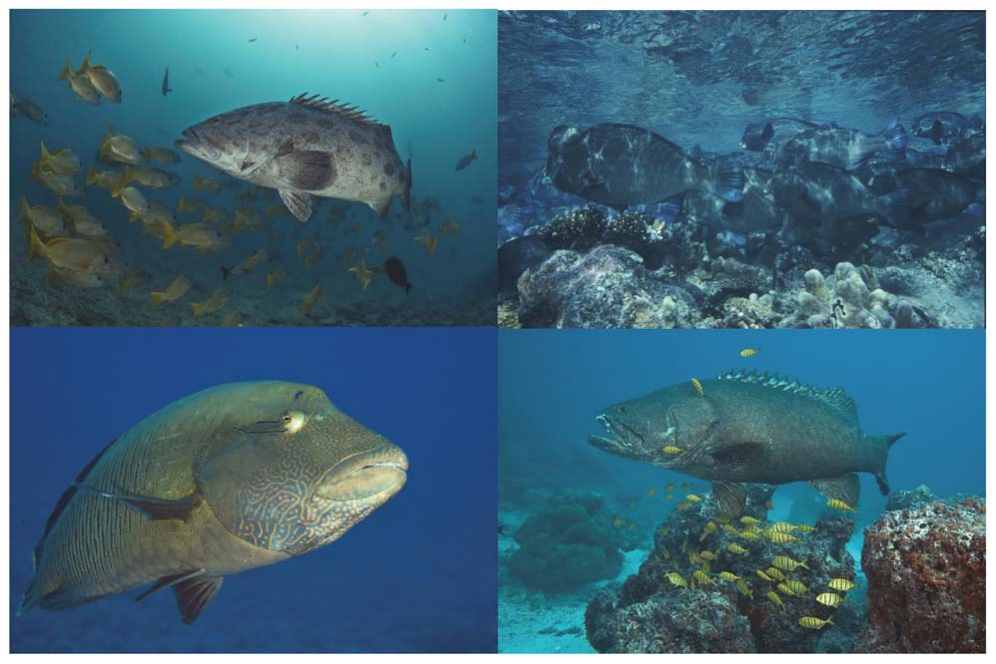
Photos of large resource fishes commonly observed at Farquhar Atoll. A. Potato cod – *Epinephelus tukula*, B. Bumphead parrotfishes - *Bolbometopon muricatum*, C. Napoleon wrasse – *Chelinus undulates*, D. – Giant grouper – *Epinephelus lanceolatus*. All photos by Peter Verhoog.

Fish assemblages at Farquhar are dominated by large predators, primarily large groupers, snappers, and jacks with large (>1 m) potato cod and marbled grouper commonly observed at many locations (Fig. 10). Several large (> 1.5 m) giant grouper (*E. lanceolatus*) were observed during the surveys and a number of commercially important grouper species are known to form spawning aggregations at Farquhar [41]. In addition, large schools of large bumphead parrotfish (one school > 600 individuals) are abundant at Farquhar, and the densities and frequencies of occurrence of this IUCN threatened species are among the highest in the world [42]. The heavy grazing pressure of this species apparently helps to keep the reef substrate nearly devoid of macroalgae, which have overgrown dead corals elsewhere in the region [30]. Bumphead parrotfish feed on a variety of benthic organisms including corals, epilithic algae, sponges, and other invertebrates and a single individual is estimated to ingest more than 5 tons of reef carbonate each year [43]; hence, even small numbers of bumphead parrotfish can have a large impact on the coral reef substrate. Likewise, the IUCN endangered Napoleon wrasse was found at over 80% of the sites, with some of the highest densities know for this species [44]. However, few sharks were observed in our survey, which is a reflection of a long history of shark fishing in the area and is likely a result of regional overfishing of these species [45]. The fish assemblage at Farquhar therefore is one with high biomass of large predators due to the extremely limited local fishing pressure but is lacking reef sharks that are typically associated with pristine reefs elsewhere around the world [21], [38], [46].

In the Caribbean, high abundance of herbivorous fishes in marine protected areas appears to reduce erect macroalgal cover and enhance coral recruitment [47]–[48]. In Micronesia, Mumby et al. [49] found high abundance of large-bodied parrotfishes was associated with low turf abundance, high coral cover, and the protection of marine reserves. These authors also found juvenile coral abundance to be higher in areas of low macroalgae cover. The large abundance and sizes of parrotfishes at Farquhar also appear to regulate erect macroalgal abundance and enhance the dominance of crustose coralline algae, which are a necessary condition for recovery of coral communities [50]–[51]. Although the conditions are appropriate, corals are not recovering quickly for a variety of likely reasons, including frequent warming events in the Indian Ocean, cyclones, and limited coral recruitment. Yet, the three-dimensional structure created by now-dead corals is still in place. The presence of live coral provides a diversity of habitats for mobile invertebrates that represent an important food source for carnivorous reef fishes; but only about 11% of reef fishes have an obligate association with corals so declines in live coral cover often have complex and indirect effects on the fish assemblage as a whole [52]. A large marine reserve in Cuba, Jardines de la Reina, has very large fish biomass with numerous apex predators such as sharks in an environment with low live coral cover, but one which still maintains the 3D reef structure [53]. A potential decline in reef fish abundance might occur at Farquhar only after the loss of the coral 3D structure [54]. It might also be that the transfer of energy up the fish food web at Farquhar, presently likely based on turf algae and plankton, is sufficient to maintain the structure and biomass of the reef fish assemblage, including large piscivores. As a matter of fact, in the 1950s large herbivorous fishes in the Caribbean fed almost exclusively on small filamentous (turf) algae when coral cover was high [55].

Our results suggest that it is possible to maintain a healthy reef fish assemblage if local threats (fishing) are reduced, which in turn will help maintain the conditions (low macroalgal cover, high CCA cover) to facilitate the recovery of corals after mortalities caused by seawater warming. In other words, reduced fishing and the establishment of no-take marine reserves may not be sufficient, but are necessary for maintaining reef resilience in the face of increasing global threats associated with climate change.

## Supporting Information

Table S1 **Check-list of Macroalgae at Farquhar Atoll.**
(DOC)
