# Supplementary material for: Coexistence of Low Coral Cover and High Fish Biomass at Farquhar Atoll, Seychelles
Source: PLoS One. 2014 Jan 29;9(1):e87359. doi: 10.1371/journal.pone.0087359 (PMC3906141; doi:10.1371/journal.pone.0087359)
Supplement: Table S1 — Check-list of Macroalgae at Farquhar Atoll. (DOC) [file pone.0087359.s001.doc]

Check-list of Macroalgae at Farquhar Atoll

1.- Macroalgae observed in this survey

2.- Macroalgae reported by Kalugina-Gutnik, A.A., Perestenko, L.P., Titlyanova, T.V., 1992. Species composition and abundance of corals and other invertebrates on the reefs of the Seychelles Islands. *Atoll Research Bulletin,* 369: 1-67.

| Taxa | 1 | 2 |
| --- | --- | --- |
| **Blue green algae** |  |  |
| *Lyngbya majuscula* (Dillwyn) Harvey |  | x |
| *Lyngbya martensiana* Meneghini |  | x |
| *Lyngbya* sp. | x | x |
| *Phormidium* sp. | x |  |
|  |  |  |
| **Red algae** |  |  |
| *Chroodactylon ornatum* (C. Agardh) Basson |  | x |
| *Stylonema alsidii* (Zanard.) Drew |  | x |
| *Erythrotrichia carnea* (Dilwyn) J. Agardh |  | x |
| *Liagora ceranoides* Lamarck |  | x |
| *Liagora* sp. |  | x |
| *Tricleocarpa cylindrica* (Ellis & Solander) Huisman & Borowitzka | x |  |
| *Tricleocarpa fragilis* (Linné) Huisman & Townsend |  | x |
| *Dichotomaria marginata* (Ellis & Solander) Lamarck | x |  |
| *Galaxaura rugosa* (Ellis & Solander) J.V. Lamouroux | x | x |
| *Gelidium pusillum* (Stackhouse) Le Jolis |  | x |
| *Parviphycus adnatus* (Dawson) Santelices |  | x |
| *Gelidiella lubrica* (Kützing) J. Feldmann & Hamel |  | x |
| *Gelidiella myrioclada* J. Feldmann & Hamel |  | x |
| *Parviphycus tenuissimus* (J. Feldmann & Hamel) Santelices | x | x |
| *Asparagopsis taxiformis* (Delile) Trevisan |  | x |
| *Hydrolithon onkodes* (Heydrich) Penrose & Woelkerling | x |  |
| *Hydrolithon gardineri* (Foslie) Verheij & Prud’homme van Reine | x | x |
| *Sporolithon ptychoides* Heydrich | x |  |
| *Melobesia* sp. |  | x |
| *Jania adhaerens* Lamarck |  | x |
| *Jania capillacea* Harvey | x | x |
| *Jania ungulata* (Yendo) Yendo |  | x |
| *Metagoniolithon stelligerum* (Lamarck) Ducker |  | x |
| *Peyssonnelia* sp. calcareous | x | x |
| *Peyssonnelia* sp. non calcareous | x |  |
| *Kallymenia* sp. |  | x |
| *Hypnea esperi* Bory |  | x |
| *Hypnea pannosa* J. Agardh | x | x |
| *Hypnea spinella* (C. Agardh) Kützing |  | x |
| *Hypnea* sp. |  | x |
| Taxa - continued | 1 | 2 |
| *Plocamium* sp. |  | x |
| *Wurdermannia miniata* (Sprengel) J. Feldmann & Hamel |  | x |
| *Ahnfeltiopsis pygmaea* (J. Agardh) P.C. Silva & DeCew |  | x |
| *Gelidiopsis* sp. |  | x |
| *Gelidiopsis scoparia* (Montagne & Millardet) De Toni | x |  |
| *Ceratodictyon spongiosum* Zanardini |  | x |
| *Botryocladia skottsbergii* (Boergesen) Levring | x | x |
| *Chamaebotrys boergesenii* (Weber van Bosse) Huisman |  | x |
| *Champia parvula* (C. Agardh) Harvey |  | x |
| *Champia viellardii* Kützing |  | x |
| *Antithamnionella* sp. |  | x |
| *Ceramium cimbricum* H.E. Petersen |  | x |
| *Ceramium fimbriatum* Setchell & Gardner |  | x |
| *Ceramium diaphanum* (Lightfoot) Roth |  | x |
| *Ceramium macilentum*  J. Agardh |  | x |
| *Corallophila apiculata* (Yamada) R.E. Norris |  | x |
| *Centroceras clavulatum* (C. Agardh) Montagne |  | x |
| *Spyridia filamentosa* (Wulfen ) Harvey |  | x |
| *Haloplegma duperreyi* Montagne | x | x |
| *Griffithsia metcalfii* Tseng |  | x |
| *Anotrichium tenue* (C. Agardh) Nägeli |  | x |
| *Dasya mollis* Harvey |  | x |
| *Amphisbetema indica* (J. Agardh) Weber van Bosse | x |  |
| *Dasya anastomosans* (Weber van Bosse) Wynne | x |  |
| *Heterosiphonia crispella* (C. Agardh) Wynne |  | x |
| *Heterosiphonia* sp. |  | x |
| *Dictyurus purpurascens* Bory | x | x |
| *Polysiphonia coacta* Tseng |  | x |
| *Polysihonia subtilissima* Montagne |  | x |
| *Polysiphonia* sp. |  | x |
| *Vidalia* sp. |  | x |
| *Lophocladia trichoclados* (C. Agardh) Schmitz |  | x |
| *Herposiphonia secunda* (C. Agardh) Ambronn f. *tenella* (C. Agardh) Wynne | | x |
| *Lophosiphonia reptabunda* (Suhringar) Kylin |  | x |
| *Lophosiphonia villum* (J. Agardh) Setchell & Gardner |  | x |
| *Chondria repens* Boergesen |  | x |
| *Chondria* sp. |  | x |
| *Chondrophycus patentirameus* (Montagne) K.W.Nam |  | x |
| *Chondrophycus parvipapillatus* (Tseng) Garbary & Harper |  | x |
| *Chondrophycus papillosus* (C. Agardh) Garbary & Harper |  | x |
| *Laurencia decumbens* Kützing | x | x |
| *Laurencia obtusa* (Hudson) Lamouroux |  | x |
| *Laurencia* sp. |  | x |
|  |  |  |
| Taxa - continued | 1 | 2 |
| **Brown Algae** |  |  |
| *Sphacelaria rigidula* Kützing |  | x |
| *Dictyopteris delicatula* Lamarck |  | x |
| *Dictyota grossedentata* De Clerck & Coppejans | x |  |
| *Padina* sp. | x |  |
| *Padina minor* Yamada |  | x |
| *Lobophora variegata* (Lamarck) Womersley | x | x |
| *Lobophora* sp. | x |  |
| *Sargassum cristaefolium* C. Agardh | x | x |
| *Sargassum ilicifolium* (Turner) C. Agardh | x | x |
| *Turbinaria ornata* (Turner) J. Agardh | x | x |
|  |  |  |
|  |  |  |
| **Green Algae** |
| *Neomeris van bosseae* Howe | x |  |
| *Caulerpa cupressoides* (Vahl) C. Agardh | x | x |
| *Caulerpa cupressoides* v. *mamillosa* (Montagne) Weber van Bosse | | x |
| *Caulerpa fastigiata* Montagne |  | x |
| *Caulerpa mexicana* Sonder ex Kützing |  | x |
| *Caulerpa mexicana* v. *pluriseriata* W.R.Taylor | x |  |
| *Caulerpa peltata* Lamarck | x | x |
| *Caulerpa racemosa* (Forsskal) J. Agardh | x | x |
| *Caulerpa racemosa* v. *macrophysa* (Sonder ex Kützing) W.R. Taylor | | x |
| *Caulerpa serrulata* (Forsskal) J. Agardh | x | x |
| *Caulerpa serrulata* f. *spiralis* (Weber van Bosse) Gilbert | x | x |
| *Caulerpa sertularioides* (Gmelin) Howe | x |  |
| *Caulerpa taxifolia* (Vahl) C. Agardh |  | x |
| *Caulerpa* sp. |  | x |
| *Caulerpa urvilleana* Montagne | x |  |
| *Chlorodesmis fastigiata* (C. Agardh) Ducker | x | x |
| *Avrainvillea amadelpha* (Montagne) Gepp & Gepp f. submersa Gepp | x | x |
| *Avrainvillea lacerata* Harvey ex J. Agardh | x |  |
| *Rhipilia tomentosa* Kützing |  | x |
| *Rhipidosiphon javensis* Montagne | x |  |
| *Udotea argentea* Zanardini |  | x |
| *Udotea orientalis* A. Gepp & E. Gepp |  | x |
| *Halimeda gracilis* Harvey ex J. Agardh | x | x |
| *Halimeda* *incrassata* (Ellis) J.V. Lamouroux | x |  |
| *Halimeda macroloba* Decaisne | x | x |
| *Halimeda macrophysa* Askenasy | x |  |
| *Halimeda micronesica* Yamada |  | x |
| *Halimeda opuntia* (Linné) J.V. Lamouroux | x | x |
| *Halimeda stuposa* W.R. Taylor |  | x |
| *Halimeda tuna* (Ellis & Solander) Lamarck | x | x |
| Taxa - continued | 1 | 2 |
| *Bryopsis pennata* Lamarck | x |  |
| *Derbesia marina* (Lyngbye) Solier |  | x |
| *Valonia aegagropila* C. Agardh |  | x |
| *Valonia fastigiata* Harvey ex J. Agardh | x |  |
| *Valonia macrophysa* Kützing | x |  |
| *Valoniopsis pachynema* (Martens) Boergesen | x |  |
| *Ventricaria ventricosa* (J. Agardh) Olsen & West |  | x |
| *Dictyosphaeria cavernosa* (Forsskal) Boergesen | x | x |
| *Dictyosphaeria versluysii* Weber van Bosse |  | x |
| *Cladophoropsis sundanensis* Reinbold | x | x |
| *Boodlea struveoides* Howe | x | x |
| *Phyllodictyon anastomosans* (Harvey) Kraft & Wynne | x | x |
| *Struvea elegans* Boergesen |  | x |
| *Microdictyon okamurae* Setchell | x | x |
| *Anadyomene wrightii* Harvey ex J.E. Gray |  | x |
| *Cladophora socialis* Kützing |  | x |
| *Rhizoclonium implexum* (Roth) Harvey |  | x |
| *Chaetomorpha linum* (O.F. Müller) Kützing |  | x |
| *Entocladia* sp. |  | x |
| *Ulva kylinii* (Bliding) Hayden et al. |  | x |
| *Ulva rigida* C. Agardh |  | x |
| *Ulva* sp. | x |  |
|  |  |  |
| **Angiosperms** |  |  |
| *Thalassodendron ciliatum* (Forsskal) den Hartog | x | x |
| *Thalassia hemprichii* (Ehrenberg) Ascherson |  | x |
